# Duration of red blood cell storage is associated with increased incidence of deep vein thrombosis and in hospital mortality in patients with traumatic injuries

**DOI:** 10.1186/cc8050

**Published:** 2009-09-22

**Authors:** Philip C Spinella, Christopher L Carroll, Ilene Staff, Ronald Gross, Jacqueline Mc Quay, Lauren Keibel, Charles E Wade, John B Holcomb

**Affiliations:** 1 Department of Pediatrics, Connecticut Children's Medical Center, 282 Washington Street, Hartford, CT 06106, USA; 2 Department of Combat Casualty Care Research, United States Army Institute of Surgical Research, 3400 Rawley E. Chambers Avenue, Fort Sam Houston, TX 78234, USA; 3 Department of Research, Hartford Hospital, 80 Seymour Street, Hartford, CT 06102-5037, USA; 4 Department of Surgery and Emergency Medicine, Hartford Hospital, 80 Seymour Street, Hartford, CT 06102-5037, USA; 5 Department of Acute Care Surgery, University of Texas Health Science Center, 6410 Fanin St, Houston, TX 77030, USA

## Abstract

**Introduction:**

In critically ill patients the relationship between the storage age of red blood cells (RBCs) transfused and outcomes are controversial. To determine if duration of RBC storage is associated with adverse outcomes we studied critically ill trauma patients requiring transfusion.

**Methods:**

This retrospective cohort study included patients with traumatic injuries transfused ≥5 RBC units. Patients transfused ≥ 1 unit of RBCs with a maximum storage age of up to 27 days were compared with those transfused 1 or more RBC units with a maximum storage age of ≥ 28 days. These study groups were also matched by RBC amount (+/- 1 unit) transfused. Primary outcomes were deep vein thrombosis and in-hospital mortality.

**Results:**

Two hundred and two patients were studied with 101 in both decreased and increased RBC age groups. No differences in admission vital signs, laboratory values, use of DVT prophylaxis, blood products or Injury Severity Scores were measured between study groups. In the decreased compared with increased RBC storage age groups, deep vein thrombosis occurred in 16.7% vs 34.5%, (*P *= 0.006), and mortality was 13.9% vs 26.7%, (*P *= 0.02), respectively. Patients transfused RBCs of increased storage age had an independent association with mortality, OR (95% CI), 4.0 (1.34 - 11.61), (*P *= 0.01), and had an increased incidence of death from multi-organ failure compared with the decreased RBC age group, 16% vs 7%, respectively, (*P *= 0.037).

**Conclusions:**

In trauma patients transfused ≥5 units of RBCs, transfusion of RBCs ≥ 28 days of storage may be associated with deep vein thrombosis and death from multi-organ failure.

## Introduction

In 2004, 29 million units of blood components were transfused in the US [1]. Due to advances in testing for infectious agents, the risk of transmitted diseases associated with blood products continues to dramatically decrease [1]. However, there are still significant risks associated with red blood cell (RBC) transfusion [2-8]. In particular, an increased volume of RBC transfusion has been associated or independently associated with adverse outcomes, including sepsis, deep vein thrombosis (DVT), multi-organ failure, and death [2-8]. A meta-analysis that included 270, 000 patients found that the risks of RBC transfusion were greater than the benefits in 42 of the 45 studies examined [9]. Additionally, a recent large prospective randomized controlled study in critically ill patients reported as a secondary outcome that in-hospital mortality was related to the amount of RBCs transfused [10].

Several investigators have attempted to determine reasons for the association between RBC transfusion and poor outcomes. A plausible biologic explanation is that lesions occurring to RBCs during prolonged storage contribute to these poor outcomes. Stored RBCs have been associated with inflammatory injury, immunomodulation, altered tissue perfusion, and impaired vasoregulation [2-6]. *In vitro *studies also document increased risk of hypercoagulation with aged RBCs [11,12]. In addition, transfusion of RBCs stored for greater than 14 to 28 days has been linked to poor outcomes [2-4,6]. However, the studies supporting the association between RBC storage and poor outcomes are mainly retrospective or prospective cohort studies, and a few studies have failed to find an association [13-18]. As a result, the theory that prolonged storage of RBCs lead to poor outcomes remains controversial [19].

We suspect that poor outcomes associated with the transfusion of RBCs stored for a prolonged period may be due, in part, to an increased inflammatory and hypercoagulable state induced by 'old RBCs' in critically ill patients. Patients with significant traumatic injuries develop a hyper-inflammatory and hypercoagulable state [20]. The pro-inflammatory and immunomodulatory nature of old RBCs [21,22] may further promote a hypercoagulable state [23,24]. DVT may be promoted in patients who are in a hypercoagulable state and multi-organ failure (MOF) is well known to occur via hypercoagulable mechanisms. We therefore hypothesized that the transfusion of old RBCs to critically ill trauma patients would be associated with an increased incidence of DVT and in-hospital mortality. A secondary hypothesis was that death secondary to MOF would be increased for patients transfused old RBCs.

## Materials and methods

This study was approved by the Institutional Review Board at Hartford Hospital, Hartford, CT, USA. We performed a retrospective cohort study of patients aged 16 years or older admitted to the Hartford Hospital intensive care unit (ICU) with traumatic injuries who received five or more units of RBCs during the hospital admission between 2004 and 2007. Patients who died in the emergency or operating room prior to ICU admission were excluded.

Data were retrospectively analyzed from prospectively populated hospital databases and patient charts. To ensure adequate follow up or to account for deaths that occurred in patients discharged prior to 180 days from admission, the social security index and Hartford Hospital databases were used to determine if there were any deaths prior to this time.

In addition to mortality, information collected included patient age, race, sex, ABO blood type, admission vital signs and laboratory values, Glasgow Coma Score (GCS), Injury Severity Score (ISS), total units of RBCs given during the entire hospitalization, plasma, apheresis platelets, cryoprecipitate, percentage of RBCs that were leukoreduced, mechanism of injury, use of DVT prophylaxis, ICU free days, and cause of death. The GCS recorded was the lower value recorded by either emergency medical providers pre-hospital or by providers in the emergency department. Race was determined by the trauma registrar and recorded in the hospital database by the following categories: white, black, Hispanic, Asian, Pacific Islander, or other. Mechanism of injury was categorized as either blunt or penetrating injury.

The incidence of DVT was determined by reviewing ultrasound results for DVT screening tests that are routinely performed on days 2 to 3 of admission for all trauma patients in the ICU. In addition to these empiric screens, if a DVT was diagnosed later in the admission due to clinical suspicion it was also included in our analysis. A DVT was defined as a thrombus that was detected by ultrasound in a deep vein. Superficial venous thrombi were not included. All forms of DVT prophylaxis were recorded including intravenous and subcutaneous heparin, subcutaneous enoxaparin, and pneumatic compression devices. The frequency of DVT prophylaxis was then compared between RBC storage age study groups. The ISS was calculated by trained staff within the Hartford Hospital Trauma Program according to the methods described by the Association for the Advancement of Automotive Medicine Abbreviated Injury Scale, 1998 Revision. Cause of death was determined by chart review and was categorized as either death due to hemorrhage, primary central nervous system (CNS) injury, or MOF. MOF was defined as two or more organ failures at the time of death. Organ failure at time of death was defined as follows: cardiac failure as requiring vasoactive agents, pulmonary failure as requiring mechanical ventilation with radiographic evidence of lung pathology, CNS failure as GCS less than 6, and renal failure as requiring dialysis or serum creatinine more than 3 mg/dl. Patients with traumatic brain injuries who remained intubated at time of death without evidence of lung injury or who were on minimal mechanical ventilator settings were determined to have died secondary to primary CNS injury and not MOF. The cause of death and organ failure at time of death was determined by chart review by a single reviewer (PCS) who was blinded to patient RBC age category and all other variables recorded for the study patients. This was accomplished by this reviewer being blinded to the database and just reviewing death certificates and patient charts. Organ failure scores such as the Sequential Organ Failure Assessment or Marshall Multi Organ Dysfunction Score were not able to be calculated from our database.

### Data analysis

We defined our study groups according to the maximum storage age of RBCs. Previous studies that used either non prestorage leukocyte reduced or prestorage leukocyte reduced RBCs have reported that RBCs above (mean or maximum) 14 to 28 days were associated with adverse events or outcomes [11,13,25-31]. Clinical studies have also reported on univariate analysis that MOF and mortality have been associated with the transfusion of RBCs of 30 and 25 days, respectively [25,26]. Therefore, as a result of our blood bank issuing RBCs that were both not prestorage leukocyte reduced and were leukocyte reduced during the time period of the study, we *a priori *decided to categorize patients according to a maximum RBC storage age of 14 or more, 21 or more, and 28 or more days. The primary groups analyzed are defined by a maximum RBC age of less than 28 days or 28 or more days, unless otherwise noted. To ensure equal amounts of RBCs transfused we matched all study groups within +/- one unit of total RBCs transfused. This was accomplished by a computerized random sampling program ("SAMPLE", SPSS, Chicago, IL, USA). The matching of patients by RBC volume was performed for each maximum RBC age analyzed (14, 21 and 28 days).

We defined study groups according to maximum RBC age, rather than mean RBC age, because the mean can obscure potential effects of older RBCs [32]. We categorized transfusion amount as 5 or more, and 10 or more units of RBCs. This was based on previous findings demonstrating that mortality dramatic increases after five or more units of RBCs have been transfused to patients with traumatic injuries [33]. To determine if there was an increased size effect with increased injury, we decided to analyze patients transfused 10 or more units of RBCs because RBC volume is associated with severity of illness [19].

The primary outcomes were DVT, and in-hospital mortality. Non-parametric and parametric data are presented as median (interquartile range) or mean (standard error of mean), respectively. The Wilcoxon Rank-sum test was used for comparison of non-parametric continuous data. The Fisher Exact or Chi Squared test was used for comparison of categorical data as appropriate. Variables with a *P *value of less than 0.1 on univariate analysis with in-hospital mortality were considered for inclusion for the multivariate logistic regression analysis. A best-fit model was determined by using changes in the log likelihood between models to determine which variables produced the most accurate model. The model with the highest chi squared statistic per degree of freedom was reported. A survival analysis at 180 days from admission was performed with a Kaplan Meier curve and Log Rank test. Statistical analysis performed with SPSS 15.0 (Chicago, IL, USA).

## Results

**Figure 1:**
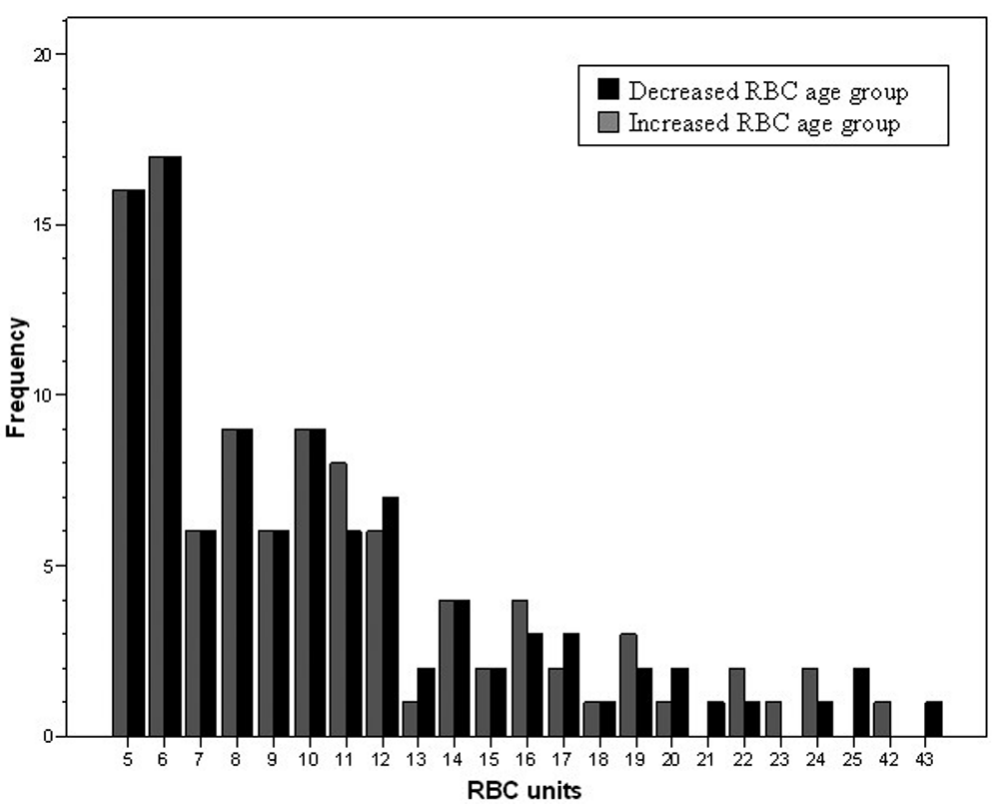
Frequency of patients transfused by total amount of RBCs for both study groups. RBC = red blood cells.

There were 270 patients identified who were admitted to the ICU with traumatic injuries and were transfused 5 or more units of RBCs. There were 202 patients who were able to be matched within 1 unit of RBC amount transfused according to the cut-off point of 28 days of RBC storage. Admission variables, ISS and outcomes were similar between the 202 patients included in the analysis and the 68 patients excluded as a result of not being able to match them with patients in the other treatment group (data not shown). In this cohort of patients who received 5 or more units of RBCs and matched by RBC amount (Figure 1), patient age, sex, race, admission vital signs and laboratory values, amount of blood products transfused, percentage leukoreduced RBCs, and ISS were similar between patients receiving RBCs of decreased and increased storage age (Table 1). Most of the patients (163 of 202 or 81%) received both prestorage leukoreduced and non-leukoreduced RBCs. There were only 39 of 202 (19%) patients who received 100% leukoreduced RBCs. The percentage of prestorage leukoreduced RBCs of all RBCs transfused was similar between RBC storage age groups (Table 1), and there was no relation between percentage of leukoreduced RBCs and mortality by chi squared analysis (Table 1) nor by logistic regression analysis with percent leukocyte reduction treated as a continuous variable (odds ratio (OR) = 1, 95% confidence interval (CI) = 0.99 to 1.01; *P *= 0.8). There were similar percentages of patients in the decreased and increased RBC storage groups who received plasma; 41.6% (42 of 101) vs 45.5% (46 of 101); platelets 17.8% (18 of 101) vs 24.8% (25 of 101), and cryoprecipitate 9.9% (10 of 101) vs 6.9% (7 of 101; *P *< 0.05). No patients in either study group received recombinant activated factor VII (rFVIIa). Blunt injury was less common in the decreased RBC storage age group compared with the increased RBC age group, 89% vs. 96%, respectively, (*P *= 0.05). Mechanism of injury was not associated with mortality on univariate analysis nor did it meet criteria for inclusion in the multivariate logistic regression analysis. The distribution of patient ABO blood group types was not similar between both study groups. Patients in the decreased RBC age group had a higher incidence of blood group type O and those in the increased RBC age group had a higher incidence of blood group type B (Table 2). No statistical differences were measured for patients with blood group types A and AB between study groups (Table 2). The maximum RBC storage age was (median, interquartile range) 19 days (16 to 24) and 34 (31 to 38) for decreased and increased RBC age groups, respectively (*P *< 0.001).

**Table 1:** Comparison of variables between patients transfused RBCs of decreased and increased storage age for patients transfused 5 or more units of RBCs

*Variables*	*Decreased RBC age group (n = 101)*	*Increased RBC age group (n = 101)*	**P *value***
**Age**	48.0 (27.0 to 60.5)	45.0 (27.0 to 63.0)	0.83
**Male%**	78/101 (77.2%)	73/101 (72.3%)	0.42
**Race (W, B, H, AP, O)%**	(76.2, 6.9, 9.9, 2.0, 5.0)	(82.2, 5.9, 8.9, 0, 3.0)	0.58
**Blunt injury**	90/101 (89.1%)	97/101 (96.0%)	0.05
**Glasgow Coma Score**	14.0 (3.0 to 15.0)	14.0 (3.0 to 15.0)	0.48
**Systolic blood pressure**	126.0 (103.0 to 141.0)	123.0 (99.3 to 143.0)	0.57
**Heart rate**	100.0 (80.0 to 120.0)	99 (79.5 to 120.0)	0.57
**Temperature (F)**	96.5 (95.6 to 97.4)	96.5 (95.2 to 98.0)	0.75
**HCO3**	21.0 (19.0 to 23.0)	21 (19.3 to 23.8)	0.81
**pH**	7.3 (7.2 to 7.4)	7.3 (7.2 to 7.4)	0.37
**Prothrombin time (seconds)**	13.0 (12.2 to 14.5)	13.2 (12.2 to 14.2)	0.91
**Hematocrit (%)**	36.9 (32.9 to 39.2)	36.1 (31.1 to 39.6)	0.32
Heparin IV (%)*	14/101 (13.9%)	19/101 (18.8%)	0.34
Heparin SC (%)*	48/101 (47.5%)	51(101) (50.1%)	0.67
Enoxaparin SC (%)*	21/101 (20.8%)	25/101 (24.8%)	0.50
Pneumatic compression device (%)*	79/101 (78.2%)	72/101 (71.3%)	0.26
**Long bone fracture (%)**	46/101 (45.5%)	48/101 (47.5%)	0.78
**Spinal cord injury (%)**	5/101 (5.0%)	10/101 (9.9%)	0.28
**RBC amount (Units)**	9.0 (6.0 to 12.5) [10.5, 6.0]	9.0 (6.0 to 12.0) [10.4, 5.9]	0.95
**RBC leukoreduced%**	50.0 (25.8 to 85.7)	62.5 (37.3 to 83.3)	0.49
**Maximum RBC storage age (days)**	19.0 (16.0 to 24.0)	34.0 (31.0 to 38.0)	< 0.001
**Median RBC storage age (days)**	14.0 (11.0 to 17.0)	20.5 (15.5 to 26.0)	< 0.001
**FFP (Units)**	0.0 (0.0 to 4.0) [2.5]	0.0 (0.0 to 4.0) [2.5]	0.82
**aPLT (Units)**	0.0 (0.0 to 0.0) [0.2]	0.0 (0.0 to 0.5) [0.2]	0.24
**Cryoprecipitate (Units)**	0.0 (0.0 to 0.0) [.1]	0.0 (0.0 to 0.0) [0.1]	0.44
**Injury Severity Score**	24.0 (14.0 to 34.0)	24 (13.5 to 33.5)	0.82

Data presented as median (interquartile range [mean] or as percentages

* indicates deep vein thrombosis prophylaxis methods prescribed

AP: Asian/Pacific Islander; aPLT: apheresis platelets; B: black; FFP: fresh frozen plasma; GCS: Glasgow Coma Score; H: hispanic; IV: intravenous; O: Other; RBC: red blood cell; SC: subcutaneous; W: white.

DVT prophylaxis was initiated in 93.1% (94 of 101) of patients in the decreased RBC age group compared with 89.1% (90 of 101) in the increased RBC age group (*P *= 0.46). There were no differences between the methods of prophylaxis between the two groups (Table 1). There were 183 of 202 (91%) of patients screened for DVT with 5 of 101 (5%) not screened in the decreased RBC age group and 14 of 101 (14%) not screened in the increased RBC age group. These 19 patients not screened for DVT had similar ISS compared with the 183 screened for DVT. Additionally, for these 19 patients without DVT screening performed, the five patients transfused RBCs of decreased storage age had similar ISS compared with the 14 patients transfused RBCs of increased storage age. ABO blood group types were similar between patients who did and did not develop DVT (*P *= 0.69; Table 2). In the 183 patients screened for DVT, the incidence of DVT was higher in the increased compared with the decreased RBC age group, 34.5% vs 16.7%, respectively, (*P *= 0.006; Table 1). The median day of DVT diagnosis was not different between increased and decreased RBC age groups, 8 days (6 to 14) vs 10 days (7 to 19), respectively (*P *= 0.58). When alternative definitions of old RBCs were used, the transfusion of one or more units of RBCs 21 or more days old was associated with increased DVT and there was an association that approached significance with the transfusion of 1 or more units of RBCs 14 or more days old (Table 3).

**Table 2:** Comparisons of ABO blood groups for study groups and outcomes measured

Blood group	Decreased RBC age group (n = 101)	Increased RBC age group* (n = 101)	- DVT (%) (n = 137)	+ DVT (%) (n = 46)	Survived (%) (n = 161)	Died (%) (n = 41)
**A (n = 72)**	38.6% (39/101)	32.7% (33/101)	37.2% (51/137)	30.4% (14/46)	34.8% (56/161)	39.0% (16/41)
**B (n = 38)**	9.9% (10/101)	27.7% * (28/101)	17.5% (24/137)	19.6% (9/46)	18.6% (30/161)	19.5% (8/41)
**AB (n = 12)**	0.0% (0/12)	11.9% (12/101)	5.1% (7/137)	10.9% (5/46)	6.2% (10/161)	4.9% (2/41)
**O (n = 80)**	51.5% (52/101)	27.7% * (28/101)	40.1% (55/137)	39.1% (18/46)	40.4% (65/161)	36.6% (15/41)

* indicates *P *value of 0.001 for comparison of ABO blood groups between decreased and increased red blood cell (RBC) age group (chi-squared test). DVT: deep vein thrombosis.

**Figure 2:**
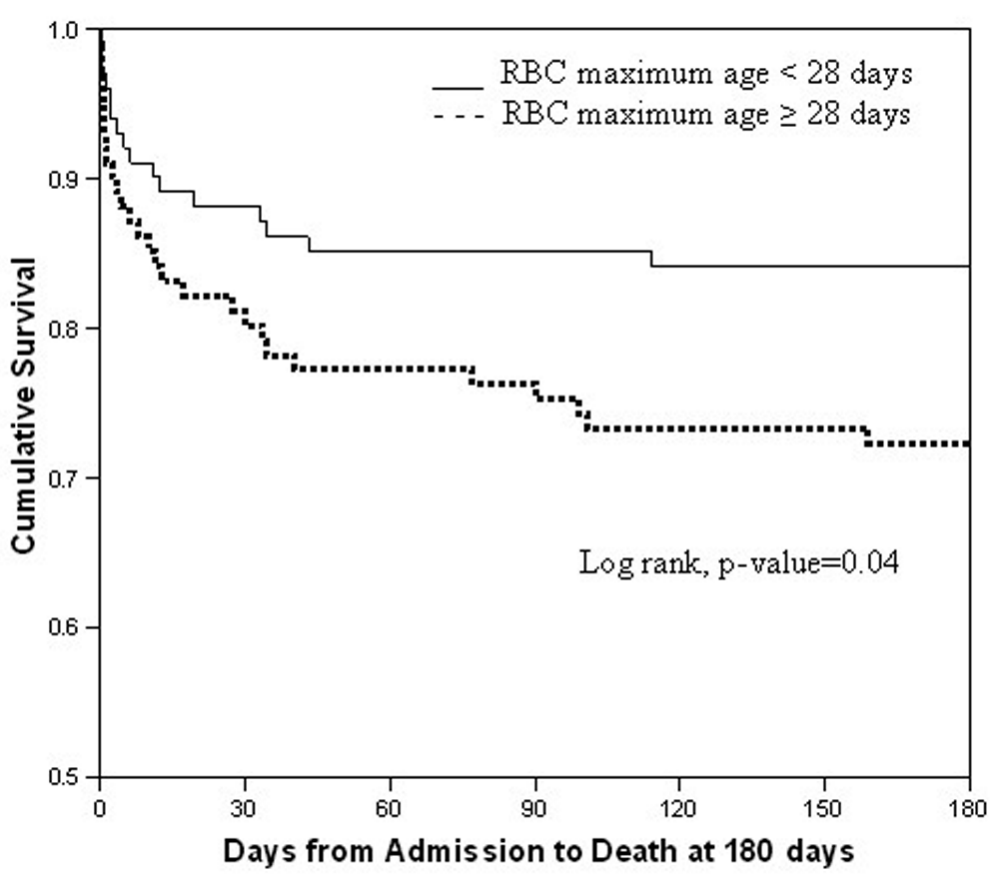
Kaplan Meier Curve of trauma associated survival over 180 days for patients transfused fresh and old RBCs. RBC: red blood cells.

**Figure 3:**
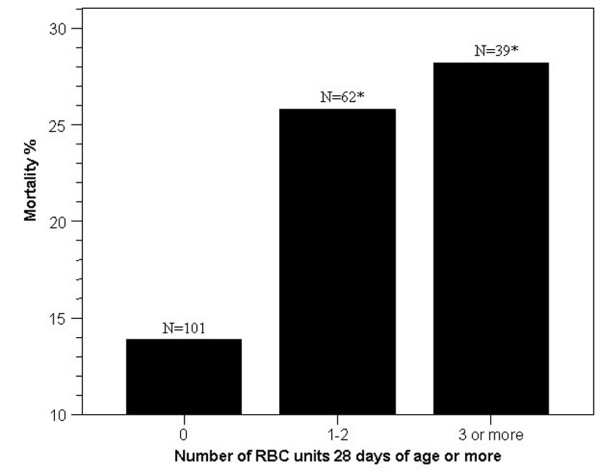
The relation between in-hospital mortality and the amount of RBC units transfused at 28 or more days of storage in patients transfused 5 or more units of RBCs. RBC: red blood cells.

In-hospital mortality was increased for those who received RBCs of increased (maximum RBC age 28 or more days) compared with decreased (maximum RBC age of less than 28 days) RBC age, 27 of 101 (26.7%) vs. 14 of 101 (13.9%), respectively (*P *= 0.02; Table 3). Additionally, patients in the increased RBC age group had an increased incidence and rate of death out to 180 days (Kaplan-Meier statistic; Figure 2). Survival rates were similar according to ABO blood group types (*P *= 0.39; Table 2). When the number of transfused RBC units 28 or more days old was analyzed to determine how many are required to measure an association with increased mortality, the transfusion of just 1 to 2 units of RBCs 28 or more days old was associated with increased in-hospital mortality (Figure 3). The mean (± standard error of the mean) ICU-free days were also increased in the patients transfused RBCs of decreased storage age compared with the increased RBC age group, 64.2 ± 2.9 vs. 54.5 ± 3.6 days, respectively (*P *= 0.036). Although the absolute mortality rate increased as the cut off of RBC age lengthened from 14 to 28 days of storage there was no statistical difference between groups when defined at 14 and 21 days of storage (Table 3).

**Table 3:** Relation of RBC storage age and outcomes for patients transfused 5 or more units of RBCs and matched for RBC amount between study groups

Outcome and maximum RBC age used to determine increased RBC age group	Patient number	Decreased RBC age	Increased RBC age	Absolute difference in outcome (%)	***P *** **value**
DVT*					
≥ 14 days	50	12.0% (3/25)	32.0% (8/25)	20.0	0.09
≥ 21 days	159	17.1% (14/82)	31.2% (24/77)	14.1	0.04
≥ 28 days	183	16.7 (16/96)	34.5% (30/87)	17.8	0.006
					
**Mortality**					
≥ 14 days	56	17.9% (5/28)	21.4 (6/28)	3.5	0.73
≥ 21 days	176	18.2% (16/88)	25.0% (22/88)	6.8	0.27
≥ 28 days	202	13.9% (14/101)	26.7% (27/101)	12.8	0.02

* see text for explanation of difference in patient numbers between number of patients analyzed for deep vein thrombosis (DVT) and mortality outcomes according to maximum red blood cell (RBC) age used to determine study groups.

On multivariate logistic regression, in-hospital mortality was independently associated with the transfusion of older RBCs for patients transfused 5 or more units of RBCs (OR = 4, 95% CI = 1.34 to 11.61; *P *= 0.01). Mortality was also independently associated with patient age, ISS, lower GCS, and total amount of cryoprecipitate transfused (Table 4). The incidence of death from MOF was increased for patients transfused RBCs of increased compared with decreased age, 16% vs 7%, respectively (*P *= 0.037; Table 5).

**Table 4:** Multi-variate logistic regression for in-hospital mortality

*Variable*	*OR (95% CI)*	**P** ***value***
**Age (Years)**	1.05 (1.01 to 1.08)	0.004
**Cryoprecipitate (units)**	12.9 (2.24 to 73.64)	0.004
**GCS**	0.89 (0.79 to 0.99)	0.04
**ISS**	1.08 (1.03 to 1.12)	0.001
**Increased RBC age group**	4.0 (1.34 to 11.61)	0.01

The area under the curve (95% confidence interval (CI)) for this regression analysis was 0.85 (0.77 to 0.92).

GCS: Glasgow Coma Score; ISS: Injury Severity Score; OR: odds ratio; RBC: red blood cell.

**Table 5:** Comparison of cause of death between study groups

Cause of death	Decreased RBC age group (n = 101)	Increased RBC age group (n = 101)	***P*** **value**
**Hemorrhage**	1/101 (1%)	1/101 (1%)	1.0
**CNS**	6/101 (6%)	10/101 (10%)	0.21
**Multi-organ failure**	7/101 (7%)	16/101 (16%)	0.037

CNS: central nervous system; RBC: red blood cell.

There were 94 patients matched by RBC units transfused who received 10 or more units of RBCs. In this cohort, there were no differences in patient age, admission vital signs and laboratory values, amount of blood products transfused, percentage of leukoreduced RBCs, and ISS between patients receiving RBCs of decreased and increased storage age (data not shown). The maximum RBC storage age (median, interquartile range) was 20 days (18 to 24) vs 34 (31 to 38) for decreased and increased RBC storage age groups, respectively (*P*< 0.001). Of the 83 of 94 (88%) patients who were screened for DVT, the incidence of DVT was higher in the increased (maximum RBC age 28 or more days) compared with the decreased RBC age group, 17 of 39 (43.6%) vs. 7 of 44 (15.9%), respectively (*P *= 0.006). Mortality was increased for those who received RBCs of increased compared with decreased storage age, 18 of 47 (38.3%) vs 6 of 47 (12.8%; *P *= 0.009). On multivariate logistic regression, in-hospital mortality was independently associated with the transfusion of RBCs of increased age (OR = 8.9, 95% CI = 2 to 40; *P *= 0.004). The incidence of death from MOF was increased in the patients transfused RBCs of increased compared with decreased age, 11 of 47 (22%) vs. 3 of 47 (6%), respectively (*P *= 0.02). The mean (± standard error of the mean) ICU-free days were raised in the decreased compared with the increased RBC age group, 59.8 ± 4.2 vs. 41.6 ± 5.2 days, respectively (*P *= 0.008)

## Discussion

This is the first study to report an independent association between the transfusion of RBCs of increased storage age (maximum RBC age 28 or more days) with increased in-hospital mortality for critically ill trauma patients transfused similar total amounts of RBCs. Death as a result of MOF was increased and ICU-free days were decreased for patients transfused RBCs of increased age. Our results also indicate that critically ill patients transfused just 1 to 2 units of old RBCs (28 or more days of storage) was associated with increased mortality. This suggests that even relatively small number of old RBC transfusions may be harmful in critically ill trauma patients. Finally, an association was measured between RBC of increased storage age (maximum RBC age 21 or more days) with DVT, which has not been previously reported.

The incidence of DVT was numerically increased with the transfusion of RBCs of 14 or more, 21 or more, and 28 or more days old with similar absolute differences in DVT incidence. Statistical significance occurred only for groups defined at 21 or more and 28 or more days of age. This is in contrast to an increasing absolute difference in mortality as the definition of old RBCs increased with statistical significance only for the groups defined at a maximum of 28 days of storage. This analysis was limited by less patients in the 14 or more and 21 days RBC age groups. In contrast, these comparisons were strengthened by the matching of the amount of RBCs transfused between all groups of increased and decreased RBC age compared in this study (≥ 14, ≥ 21, and ≥ 28 days).

**Figure 4:**
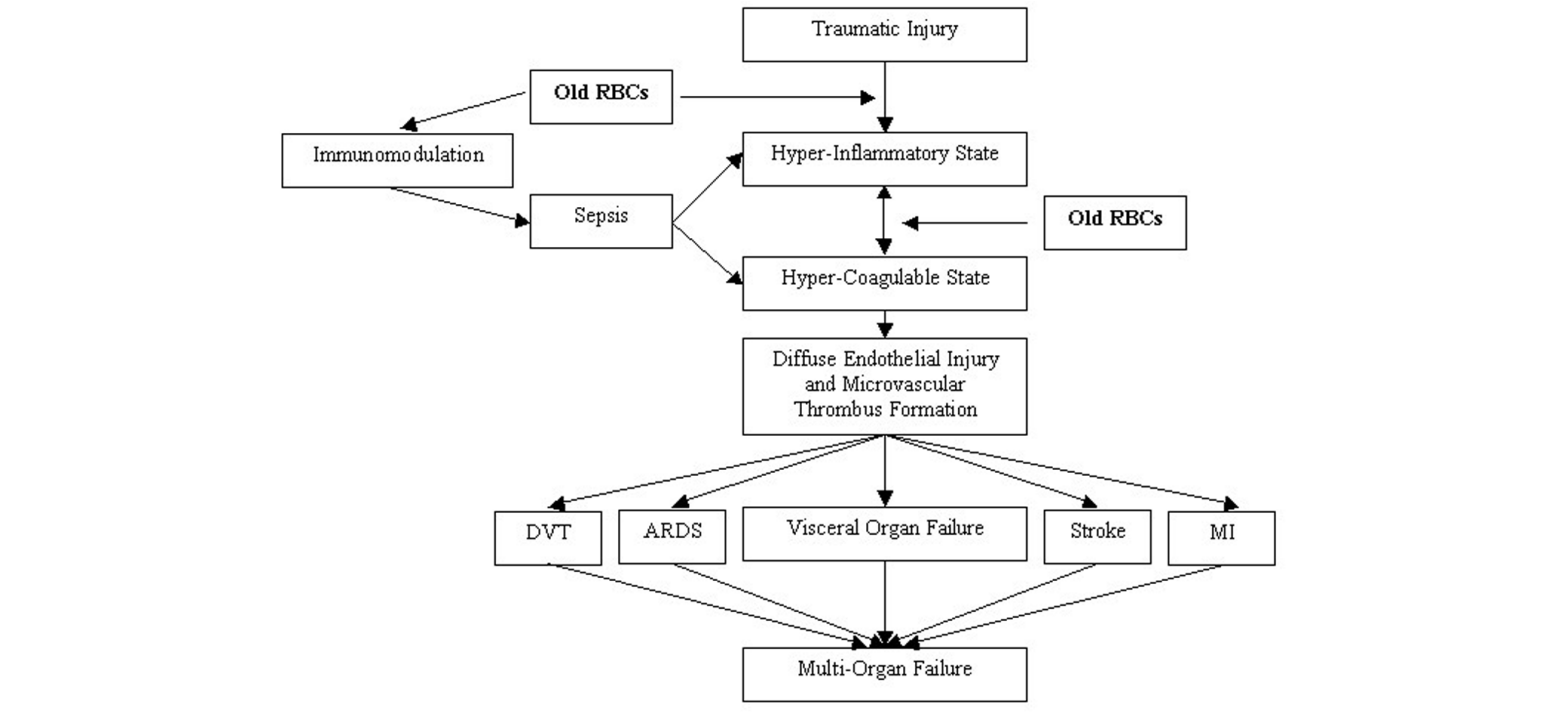
Flow diagram of describing potential mechanism of how old RBCs increase risk of multi-organ failure via inflammatory and coagulation pathways. ARDS: acute respiratory distress syndrome; DVT: deep vein thrombosis; MI: myocardial infarction; RBC: red blood cells.

Although our study was not designed to investigate mechanisms associated with findings, our hypothesis was based on previous literature reviewed by Park and colleagues regarding the interplay between inflammation and hypercoagulation [20] and the literature supporting old RBCs are hyper-inflammatory, immunomodulatory, and impair microvascular perfusion and vasoregulation [2-6]. Old RBCs have been demonstrated to increase polymorphonuclear cell activation, superoxide anion and IL-8 concentrations [21,22], which may be a result of pro-inflammatory bioactive lipids, which increase with RBC storage time [5,34]. In fact, bioactive lipids that accumulate with storage time have recently been associated in a laboratory study with increased thrombin generation. In these prestorage leukoreduced RBCs increased thrombin generation occurred after 31 days of storage in AS-1 solution [12]. Another recent publication indicates RBC storage time is associated with increased generation of procoagulant phospholipids [11]. Immunomodulation and increased risk of sepsis independently associated with old RBCs will also increase these risks [25,27,35-38]. We theorize that the hyper-inflammatory and hypercoagulable state associated with trauma is potentiated by the pro-inflammatory and immunomodulatory effects of old RBCs [21,22,35,37,39], which then increases the risk of DVT and death as a result of MOF via hypercoagulation and diffuse endothelial injury (Figure 4).

We compared ABO blood group types between study groups because a previous report indicates that patients with type A blood have increased concentrations of factor VIII and von Willebrand's factor, which was associated with increased risk of DVT [40]. In our analysis, although there was not an equal distribution of patient ABO blood groups between study groups, we did not measure any relation between patient blood type and incidence of DVT or in-hospital mortality. Therefore, although it is important to determine the effect of ABO blood type on risk of thromboembolic events in future analyses, there was no apparent effect on either DVT or mortality in our study.

Previous studies reported an independent association between the transfusion of old RBCs and increased risk of sepsis, MOF, and death in all types of critically ill patients [25-27,32,41,42]. However, a consistent criticism of some of these studies is that there were not equal amounts of RBCs transfused in the study groups. The concern regarding the amount of RBCs transfused per study group is related to the concept that RBC amount itself strongly correlates with injury severity and can never be adequately adjusted for with multivariate logistic regression [9]. Our findings may have increased validity compared with previous studies as a result of our method of specifically matching patients by the amount (± 1 units) of RBCs transfused. Previous reports have also indicated that RBC transfusion volume was associated with DVT [7,8]. These studies did not take into account the storage age of RBCs.

The recent study by Weinberg and colleagues in trauma patients analyzed the number of units greater than 14 days of age and reported that for patients transfused 6 or more units of pre-storage leukoreduced RBCs that the odds ratio for death was increased for those who were transfused 1 to 2 units greater than 14 days old and that the odds of death were higher for patients who were transfused 3 or more units of RBCs [32]. Although this study did not compare patient groups that specifically matched patients by amount of RBCs, their findings are consistent with the present study results. Not only is there consistency with increased mortality in patients transfused old RBCs there is also consistency in that both studies demonstrate it only takes 1 to 2 units of old RBCs to increase the odds of death and the size of the effect is greater for patients with increased injury indicated by the amount of RBCs transfused. In our analysis patients transfused 10 or more units of RBCs had an approximate doubling of the OR for mortality when compared with patients transfused 5 or more units of RBCs.

A major difference in our report compared with the study by Weinberg and colleagues is our definition of when RBCs become 'old'. The different methods of defining old RBCs used in various studies have made comparing results problematic. Previous definitions have included mean, median, and maximum RBC storage age in addition to the number of RBCs transfused above 14 and 21 days of age [25-27,32,41]. The change from the use of non-prestorage leukocyte reduced RBCs to the transfusion of prestorage leukocyte reduced RBCs has also made it difficult to determine the optimal definition of old RBCs. Independent associations with the amount of non-prestorage leukocyte reduced RBCs more than 14 and 21 days old have been reported with sepsis [27]. The mean RBC storage age and the amount of non-prestorage leukocyte reduced RBCs of more than 14 and 21 days old have also been associated with increased MOF [26]. Optimally when comparing the effect of RBC storage age on outcomes the definition of fresh and old RBCs should not allow for mixing of RBC storage age between groups as was done in the study by Koch and colleagues [41]. In this study of more than 6000 patients, the fresh RBC group was defined as those who were only transfused RBCs of 14 days of storage or less and the old RBC group received only RBCs of greater than 14 days of storage. In smaller retrospective studies that are not large enough to have complete separation of fresh and old RBCs, it is more appropriate to use the maximum RBC age transfused than mean RBC age to define if the patient received fresh or old blood. This is because the adverse effects of RBCs have been measured with relatively small amounts transfused [10,32,41]. When mean RBC age is used to define patients who received fresh or old RBCs this method allows for the youngest RBCs to balance out or negate the contribution of storage age from the oldest unit transfused. For example a patient who receives 2 units at 40 days old and 8 units at 10 days old will have a mean of 16 days whereas for a patient who receives 10 units at 16 days of storage the mean will be 16 days. The patient transfused 2 units at 40 days would theoretically be at increased risk but using the mean RBC age to define fresh vs old RBC patient groups does not identify this difference whereas using maximum RBC age does.

Additionally, an individual patient's severity of illness may influence the clinical effect of old RBCs. It is our theory that the most critically ill patients will be most affected by older RBCs. This concept is supported by the study performed by Weinberg and colleagues [32], where patients who were sicker had increased OR with mortality with 'old' RBCs, and also in our study where patients transfused 10 or more units of RBCs had an increased OR for mortality compared with patients with decreased injury who only received 5 or more units of RBCs.

A source of criticism of previous studies is the inclusion of patients who received non-leukocyte reduced RBCs. A recent study by Weinberg and colleagues only included patients who received prestorage leukoreduced RBCs and their results still demonstrated an increased risk of death with the use of RBCs more than 14 days of storage for patients transfused 6 or more units of RBCs [32]. In addition, while our study groups received a mixture and similar proportions of both non-leukoreduced and leukoreduced RBCs, the percentage of leukoreduced RBC units was not associated with survival on univariate or multivariate logistic regression analysis.

The clinical benefits of prestorage leukoreduced RBCs are controversial. Although there are some benefits to their use [43], it is our belief that universal leukoreduction cannot mitigate all the adverse effects of prolonged RBC storage in critically ill patients. For example, the deformability and nitric oxide mechanisms will very likely not be altered by leukoreduction nor will the proinflammatory effects of bioactive lipids that increase with storage time [34]. As has been suggested previously, perhaps the routine use of RBC washing for patients at risk of inflammatory and immunomodulatory injury should be considered when old RBCs need to be transfused [5]. The evidence that only one seven minute wash cycle is required to mitigate the proinflammatory effects of old RBCs [21] and the current development of large multi-unit RBC washing devices may make this approach a more viable option for patients requiring a large amount of RBCs rapidly.

During the time period of the study the typical practice at our trauma center did not include the frequent early use of plasma, platelets and cryoprecipitate as is described in Table 1 and there was no use at all of rFVIIa. Despite the low frequency of the use of these blood products the amount of cryoprecipitate was independently associated with in-hospital mortality. A potential explanation for these findings is that the use of cryoprecipitate was used very late in the resuscitation of patients when the patient was already in a state of irreversible shock and high risk of death secondary to hemorrhage. These findings are in contrast to recently published US military data indicating that the early use of procoagulant blood components to include cryoprecipitate and plasma are independently associated with improved survival [44-46]. As we are unable to determine when specifically cryoprecipitate was transfused in our study, we cannot easily explain these results.

Our study was limited primarily by its retrospective nature. As such, limitations include possible selection bias and the potential for not adequately adjusting for unmeasured confounding variables. As a result our findings can only be hypothesis generating and are not intended to be interpreted as hypothesis testing. A significant limitation of our study was the inability to match RBC volume according to the timing of RBCs transfused. RBCs transfused after the development of DVT could not have influenced the development of DVT, although this risk should be equal in both RBC age groups studied. However, storage age of RBCs administered to the patients in this study was not chosen specifically by anyone. The age of RBC administered was according to blood bank policy and was consistent during the study period. Therefore, the risk of selection bias regarding the age of RBCs transfused is small. Although we did not include all potential confounders such as time from injury to operative control of bleeding, ICU practices etc., we were able to include a large number of variables that have been associated with mortality in trauma and our regression analyses were strong according to the high area under the curve measured in the model. The only difference noted in the primary patient population was an increased incidence of penetrating injury in the decreased RBC age group. However, the mechanism of injury was not associated with mortality and therefore is not a confounding variable on RBC age and mortality. Another potential limitation is that DVT screening did not occur for all patients included in the study. DVT screening occurred in 91% of patients included. Some may have been transferred out of the ICU before it was ordered, some may have died before it could be performed, and others may not have received one due to physician error in not ordering one. The timing of DVT screening was also not uniform or standardized. This may have introduced sampling bias. Although the use of each method of DVT prophylaxis method was similar between study groups, the inability to compare the timing of DVT prophylaxis initiation from admission is another limitation. Finally, our analysis of DVT was limited by our inability to adjust for confounding variables.

As the literature continues to demonstrate that older RBCs are potentially harmful in critically ill patients, and there is biologic plausibility, consistency, and size effect, well-designed prospective controlled trials to test this hypothesis must be performed. The clinical effects of the storage lesion and the precise mechanisms of how they potentially cause adverse effects need further study. Blood banks do not routinely record the storage age of RBCs transfused. This needs to become standard to facilitate the study of age of RBC on outcomes. Blood banking methods or alternative storage solutions also need to be studied to determine if these potential adverse effects can be mitigated. Furthermore, the criteria for licensing current and future storage solutions should also include the monitoring or testing of the many potential adverse effects of the storage lesion. Finally, study is needed in human subjects to determine if stored RBCs are able to perfuse the microvasculature tissue and increase oxygen delivery and consumption for critically ill patients with shock.

## Conclusions

In trauma patients transfused 5 or more units of RBCs, DVT, and in-hospital mortality was increased with the transfusion of old RBCs when compared with a group of patients of similar severity of injury who were transfused RBCs of decreased storage age. After adjustment for other variables associated with mortality there was an independent association with the transfusion of older RBCs with in-hospital mortality. The increased risk of mortality was associated with the transfusion of just 1 to 2 units of RBCs greater than 28 days of storage and could be accounted for by increased MOF. As there is no evidence that RBCs of increased storage age improve microvascular delivery of oxygen and consumption for patients in a shock state and there is a substantial amount of evidence that indicates they may increase injury in critically ill patients, the preferential use of fresh RBCs can be appropriate if local inventory allows for this without substantially increasing RBC waste. Prospective randomized study in this population is needed.

## Key messages

For patients with traumatic injuries transfused 5 or more units of RBCs and alive on ICU admission:

• DVT is associated with the transfusion of RBCs of 21 or more days of storage in this study population.

• Mortality at 30 days is independently associated with the transfusion of RBCs of 28 or more days of storage in this study population.

• Mortality at 30 days was increased with 1 to 2 units of RBCs of 28 or more days of storage in this study population.

• This is the first study to specifically match study groups by the volume of RBCs transfused, which eliminates the potential confounding effect of RBC volume transfused on DVT and mortality.

• Future studies should investigate the potential affect of RBC storage age on thrombotic mechanisms.

## Abbreviations

CI: confidence interval; CNS: central nervous system; DVT: deep vein thrombosis; GCS: Glasgow Coma Score; ICU: intensive care unit; IL: interleukin; ISS: Injury Severity Score; MOF: multi-organ failure; OR: odds ratio; RBC: red blood cell; rFVIIa: recombinant activated factor VII.

## Competing interests

No conflict of interests existed with any of the co-authors and the data presented in this study. The primary author (PCS) had full access to all of the data in the study and takes responsibility for the integrity of the data and the accuracy of the data analysis. The views and opinions expressed in this manuscript are those of the authors and do not reflect the official policy or position of the Army Medical Department, Department of the Army, the Department of Defense, or the United States Government.

## Authors' contributions

PCS contributed to study design, data analysis, and manuscript preparation and obtained funding. CC contributed to study design and manuscript preparation. IS contributed to study design, data analysis and manuscript preparation. RG contributed to study design, and manuscript preparation. LK contributed to data collection and manuscript preparation. CEW contributed to study design, data analysis and manuscript preparation. JBH contributed to study design, data analysis and manuscript preparation. All authors read and approved the final manuscript.
